# Mental Health Conceptualization and Resilience Factors in the Kalasha Youth: An Indigenous Ethnic and Religious Minority Community in Pakistan

**DOI:** 10.3389/fpubh.2018.00187

**Published:** 2018-07-17

**Authors:** Fahad R. Choudhry, Tahir M. Khan, Miriam Sang-Ah Park, Karen J. Golden

**Affiliations:** ^1^Department of Psychology, Jeffrey Cheah School of Medicine and Health Sciences, Monash University, Bandar Sunway, Malaysia; ^2^National Institute of Psychology, Quaid-i-Azam University, Islamabad, Pakistan; ^3^Institute of Pharmaceutical Science, University of Veterinary and Animal Sciences, Lahore, Pakistan; ^4^School of Social and Health Sciences, Leeds Trinity University, Leeds, United Kingdom

**Keywords:** indigenous, resilience, ethnic minority, mountain people, marginalized, mental health, South Asia, Pakistan

## Abstract

The Kalasha are a religious, ethnic, and linguistic minority community in Pakistan. They are indigenous people living in remote valleys of the Hindu Kush Mountains in northern Pakistan, neighboring Afghanistan. The Kalasha are pastoral, as well as agricultural people to some extent, although they are increasingly facing pressures from globalization and social change, which may be influencing youth and community development. Their traditional world view dichotomizes and emphasizes on the division of the pure (*Onjeshta*) and the impure (*Pragata*). There remains a scarcity of literature on mental health and resilience of indigenous communities in South Asia and Pakistan generally, and the polytheistic Kalasha community specifically. Thus, the current study was conducted with the aim to explore the cultural protective factors (resilience) of the Kalasha youth (adolescents and emerging adults) and to explore their perceived etiological understandings and preferred interventions for mental health support systems. The theoretical framework of Bronfenbrenner's (1, 2) ecological systems model was used. Interpretative Phenomenological Analysis (IPA) was conducted, considering the advantage of its idiographic approach and the “double hermeneutic” analytic process. This methodology was consistent with the aim to understand and make sense of mental health and resilience from the Kalasha indigenous perspective. A total of 12 in-depth interviews were conducted with adolescents and emerging adults (5 males, 7 females), along with ethnographic observations. The analysis revealed 3 superordinate themes of mental health perceptions and interventions, each with more specific emergent themes: (1) Psychological Resilience/Cultural Protective Factors Buffering Against Mental Health Problems (*Intra-Communal Bonding & Sharing; Kalasha Festivals & Traditions; Purity Concept; Behavioral Practice of Happiness and Cognitive Patterns*); (2) Perceived Causes of Mental Health Issues (*Biological & Psychosocial; Supernatural & Spiritual*; *Environmental*); and (3) Preferred Interventions [*Shamanic Treatment; Ta'awiz (Amulets); Communal Sharing & Problem Solving; Medical Treatment; Herbal Methods*]. The overall findings point to the need for developing culturally-sensitive and indigenous measures and therapeutic interventions. The findings highlighted the Kalasha cultural practices which may promote resilience. The findings also call for indigenous sources of knowledge to be considered when collaboratively designing public health programs.

## Introduction

The Kalasha are an indigenous marginalized group settled in the northern mountains of eastern Afghanistan and North-West Pakistan. They are a pagan tribe that practice their own tradition and culture (3). Their religious beliefs are polytheistic and animistic. Some of the Kalasha believe that their roots are in Greece and they are the descendants of Alexander the Great and his army. However, the archaeological research through excavation and exploration in Chitral revealed that Kalasha were present in Chitral even before the arrival of Alexander the Great in this region (4, 5). Historically, Kalasha settlements were spread over the Chitral district in Pakistan and Nuristan in Afghanistan, but now they have been shrunken and limited only to three small valleys of Chitral, Pakistan (6–8). The Kalasha belong to an oral tradition, without having any written scripture about their religion and culture (9). The Kalashamon (“Kalasha language”), is one of the many threatened and near extinction languages of the world (10, 11). In the recent years, however, the linguistic community has started making some efforts to save the Kalasha language through documenting the text (12).

The Kalasha world, or their “fundamental dichotomisation of the natural environment” (13), is divided into two spheres of ónǰeṣṭa (pure) and prágaṭa (impure). The ónǰeṣṭa (pure) spheres include the heights, the mountains, gods, wildlife and goat stables, mountain spirits, fairies and the space between the back wall of the houses and the fire space in the middle (14). The prágaṭa, on the other hand, represents the places at the bottom of the valleys that includes the graveyard and Bashali (a place where women stay during menstruation and during childbirth) (14–17). Ónjeṣṭa also includes animals. For example, goats (as they graze at high altitude in mountains) are pure, while the chickens and the eggs are considered prágaṭa (impure). The neighboring non-Kalasha communities are thus settled in the lower portion of the valleys and come under prágaṭa spheres. The Kalasha community, therefore, settled in an intermediate space between the dichotomy of prágaṭa and ónǰeṣṭa (14, 17).

The positive connotation in ónǰeṣṭa is not related to a blissful life in the hereafter. Also, although prágaṭa doesn't have a positive connotation, it has nothing to do with sin or the devil (5). Additionally, there is no association of these two poles with sexual morals, cleanliness or devotion, like the rest of the majority of the Pakistan community's view of “pure/impure” (18). Nowadays, the Kalasha have started talking about hell and heaven and often relate this to the myth of Adam and Eve owing to the influence of Islam (5, 19). However, the traditional Kalasha vision, rooted in their religious system, has limited concern for the hereafter or the idea of a prize or a punishment in the life after death (5).

The Kalasha are an agro-pastoral community (20). Their economy is based on pasture and agriculture historically (21). However, their agriculture is very small scaled and the pastoral economy has been endangered because of the symbolic significance of the pastures in their cultural traditions (20, 21). For example, goats are culturally important, intrinsically sacred, and pure animals amongst the Kalasha because of their association with wild animals of the mountain region (20). Even on festival dances, playing of goats and clashing of their horns during combats is enacted. Goats are slaughtered and their blood is believed to provide protection against evil forces (22, 23). Contrary to the role of cattle and sheep in pastoral subsistence, the obsession of the Kalasha with goats can be seen in multiple ways (13). Goats' symbolism can be seen in entire Kalasha religious culture such as goat carvings on doors; pillars of houses; altars, and images on walls of clan temples (24). The images of goats are also molded in dough or imprinted on bread at important rituals (25).

The Kalasha are surrounded by a conflict driven Afghanistan where an extreme and hard-line religious narrative has been built since the 1980s and the narrative kept on spreading to the neighboring places including parts of Pakistan (26). The same narrative forced the whole Kalasha community restricted to three valleys, however, once they were settled in the Nuristan district of Afghanistan in the past. The counter-narrative needs to be built very timely in order to preserve the diminishing cultural heritage of Kalasha.

The Kalasha are a vulnerable and endangered community considering their challenging geographical location, and security threats and their youth are constantly striving to preserve their unique culture (3). Therefore, in order to gain an in-depth understanding of an individual's protective factors, there is a need to understand the cultural conceptualization of resilience and the role of ecological processes in development of an individual (27). There is a need to look deeper into the ecological system owing to the influence of the environment of an individual on his/her development (27, 28). According to Bronfenbrenner's ecological system model, an individual is surrounded by layer of contexts under influence of multidirectional factors (e.g., family, environment, and context) (1). The predominant focus is on the multidirectional interplay of environment, context, and ecological system of an individual (29). According to Bronfenbrenner, there are five interlinked levels in the ecological environment of a person: microsystem (i.e., family and school etc.); mesosystem (interaction between two systems); exosystem (system that has an indirect influence on a person, e.g., parent's workplace); macrosystem (community level norms, beliefs and culture); and chronosystem (the chronological component) (2). Furthermore, Putnam (30) highlighted the concept of social capital which includes community networks, sense of belonging, civic engagement, and trust in community are among the important factors contributing to social capital.

Most of the research studies on mental health and resilience have been conducted in high-resourced developed countries (27, 31, 32). Furthermore, very limited research is conducted in low-resourced countries to understand the responses of minority groups regarding mental health issues (31). More indigenous resources, interventions, and methods can be developed if researchers explore the cultural, contextual, bioecological protective factors and contextually and culturally sensitive intervention models (31). Therefore, the current study was conducted with the objective of understanding the educated young Kalasha's mental health conceptualization, treatment preference and their indigenized cultural protective factors against mental health issues.

## Methods

### Study design and authors

The study was conducted using the overarching framework of Bronfenbrenner's ecological system (29). The first author was a non-Kalasha researcher who had worked with the marginalized communities of Pakistan for the past 6 years, and had knowledge of the local dynamics and culture of indigenous communities of Pakistan. The first author has been trained as a psychological researcher and served in multiple sectors (public, private, and non-government developmental segments). The first author has vast experience using qualitative research, more specifically, he has 7 years of experience with Interpretative Phenomenological Analysis (IPA). The first author along with three local researchers visited the Kalash valleys for data collection.

Interpretative Phenomenological Analysis (IPA) is a qualitative analysis approach which specifically explores people's sense making of their experiences with predominantly psychological interest (33). In IPA, the researcher carries out in-depth, extensive, first-person interviews of research participants. The researcher believed in decolonizing research methodologies, therefore, he took an initiative to foster local community connections, for participatory research with the indigenous Kalasha community. The co-authors of the current study are also experienced researchers with ample understanding of indigenous and qualitative methodologies.

For the current study, we adhered to the principles and guidelines of IPA (33). As IPA is an inductive approach, we framed our research question ensuring flexibility. We tried to maintain quality, trustworthy data collection, analysis, and writing up of this research project by implementing principles of IPA. The team members had regular discussions and team members explicitly monitored the research process to ensure that the principles of IPA were followed. Participants were recruited from the three valleys of Kalasha through snowball sampling. Twelve Kalasha people were interviewed for this IPA study.

### Research paradigm/epistemology

The epistemology of IPA is experiential and hermeneutic. In IPA, the interest is to understand an individual's connection to the world (and to the things in it which matter to him/her) through that individual's meaning-making (34). The assumptions of IPA are: developing an understanding of the world necessitates an understanding of the experiences; and the IPA researchers are involved in the personal narratives of the individuals who are “always-already” engrossed in a linguistic, cultural and physical world (34). Therefore, we took an idiographic approach to our research project, for enabling an in-depth focus on the particular individuals of the Kalasha sample. In IPA, the researchers do not directly access experiences from narratives, rather a process of intersubjective meaning-making helps them in accessing a phenomenon. For immersing in participants' experience, the IPA researchers need to be able to recognize and reflect upon their own experiences and assumptions.

### Participants

The research participants we recruited formed a homogenous sample of the Kalasha youth, struggling for preservation of their unique culture, hence, the research question of understanding their resilience was meaningful. As the mode of enquiry in IPA is idiographic and inductive, a closely-defined group of participants represents a certain group or perspective in the area of study rather than a population is needed. The recruitment of a homogenous sample of participants assisted in providing insights into the similarity and variability of people representing that perspective. Recruitment and selection of the research participants was carried out through the snowball sampling method, as after the first few interviews we requested the participants to refer to their friends (who represent Kalasha youth) for participation in this study. The sample consisted of young Kalasha; from Bhamburat and Birir valleys. All participants were adolescents and emerging adults; identified themselves as Kalasha (5 men, 7 women); were born and had lived in Kalasha and, were fluent in the Urdu language-commonly spoken in Kalasha valleys. Most of the participants were enrolled to undergrad degree and/or post-graduate level. Majority of the participants were students, however, one participant was a part time student and also worked in the fields and another was a part-time student. The Kalasha do understand and speak Urdu (35, 36), therefore, interviews were conducted in the Urdu language. A detailed analysis of a total of 12 in-depth interviews of 12 participants was carried out. The participants' age ranged from 18 to 26 years thus they are considered the youth of Kalasha community.

### Data collection

The data were collected in the form of in-depth semi-structured interviews. The time duration of these interviews varied from 55 to 140 min. On average, each interview took 97 min. The first author interviewed all the participants and other researchers helped in handling recording devices and taking field notes. This study was carried out in accordance with the recommendations of the ethics guidelines of the Monash University Human Research Ethics Committee (MUHREC) and the ethics committee of the Punjab Institute of Mental Health (PIMH). The protocol was approved by the MUHREC (MUHREC; CF15/4575 - 2015001970) and the Ethics Committee, PIMH. All subjects gave written informed consent in accordance with the Declaration of Helsinki. All participants were informed that they were free to stop the interview or withdraw their data, should they wish. We used the interview schedule flexibly according to IPA good practice (33).

### Procedure

Semi-structured interviews were carried out in the Urdu language in order to collect the data. The research participants were asked to talk about their experiences of mental health and protective factors against mental health problems. An interview schedule guided the researchers, but the participants were allowed to talk freely about their own experiences. The researcher spontaneously asked questions in response to the participants' accounts. All the interviews were carried out at participants' homes on a one-to-one basis. The arrangements were done to conduct the interviews in a separate room of the participants' houses in order to ensure confidentiality of the responses. The interviewer probed the important and interesting issues brought forth by the participants themselves. All the interviews were audio-recorded on two separate audio-recorders. The recordings of all the interviews were transcribed verbatim and then translated into English. The field notes were taken during the interviews by the accompanying researchers. All the data was anonymized to ensure confidentiality. The translated interviews were checked by two language experts who had command over both languages (i.e., Urdu and English).

### Data analysis

In order to carry out the analysis, we followed the IPA procedures outlined by Smith et al. (33). IPA is committed to the idiographic approach—a process of analysis which begins with a detailed assessment of each case followed by a search for similar responses across cases (37). Both convergence and divergence is carried out in the analysis by reading the transcripts line-by-line. The analysis proceeds by assessing the points of descriptive, linguistic, and conceptual notes throughout. The initial step of IPA comprises developing an open mind and an exploratory attitude for producing a comprehensive and in-depth account of the data (33, 38). In the other margin, the researcher transformed the initial notes into emergent experiential themes. In a resonant and concise way, the themes set out to grasp the main aspects of each participants' experience framed by the interpretations of the analyst. Using techniques of abstraction and subsumption (33) within case and across the participants, themes were grouped and superordinate themes were produced. After producing a list of superordinate themes and contributory subthemes with reference pointers to the supporting evidence in the interview transcript for the first case, the process was repeated for every case. In the end, the 12 individual tables were reviewed at the same time, major common themes, and points of strong convergence and divergence were highlighted.

The superordinate themes were then transformed into a narrative account with analysis supported by verbatim extracts from every participant. The main author took the lead on the initial analytic steps and at each stage the data was audited by co-authors. Through this rigorous search, any kind of the influence of assumptions on the analysis was minimized. This auditing led to minor modifications. In the later stages of analysis, writing was done in a collaborative way and ideas were shared, challenged and modified. All the co-authors contributed to the writing up and gave significant comments on interpretations. This method is consistent with the good practice in hermeneutic phenomenology and helps to guarantee rigor. Specifically, we adhered to Gadamer's (39) affirmation that important aspects of the reflexive interpretative arc only become apparent during analysis and good analysis incorporates new insights in the write up (40). The raw data of the present study were the transcripts of the interviews.

## Results

The IPA was conducted to extract themes from the data. We have extracted a total of 3 superordinate themes, each with emergent themes: (1) Psychological Resilience/Cultural Protective Factors Buffering Against Mental Health Problems (Intra-communal Bonding & Sharing; Kalasha Festivals & Traditions; Purity Concept; Behavioral Practice of Happiness and Cognitive Patterns); (2) Perceived Causes of Mental Health Issues (Biological & Psychosocial; Supernatural & Spiritual; Environmental); and (3) Preferred Interventions [Shamanic Treatment; Ta'awiz (Amulets); Communal Sharing & Problem Solving; Medical Treatment; Herbal Methods]. Each major category of theme or superordinate theme with emergent subthemes is discussed in the following section.

### Psychological resilience/cultural protective factors buffering against mental health problems

The Kalasha youth discussed certain factors that they considered as their cultural protective factors against mental health issues. Here, it is noteworthy that the term “mental health problems” is generally a broad term and used intentionally in order to understand Kalasha youth's understanding of “mental health.” Therefore, in this result section, when we use the term psychological resilience or cultural protective factors buffering against mental health problems, it refers to neurotic as well as psychotic domains of the problem. However, we have observed a pattern in every interview where a participant started sharing his/her views regarding protective factors and initially conceptualized “mental health” as psychotic phenomenon and then with the passing conversation and probing from the interviewer, the participant also shared his/her views of resilience factors against “less severe” mental health issues, and/or the symptoms of common mental disorders, like disturbed sleep, decreased appetite, irritability, and sadness.

#### Intra-communal bonding and sharing

The intra-communal bonding and sharing culture among Kalasha youth was the most frequently discussed factor that contributes to their well-being and serves as a cultural protective factor against mental health issues (both minor and severe). It is pertinent to note that in the very first theme of cultural protective factors, the young Kalasha participants reflected upon their shared value of unity and membership of their traditional culture. They do not only own their culture with pride, but also consider their communal bonding and culture of generosity as important factors that make them resilient. At the conceptual level, their positive identification with sharing and their strong bonding culture shows their contentment with their cultural identity. The following excerpts from participants' interviews reflect their intra-communal bonding and sharing:

“*Kalasha are our own people and we live together, when there is any problem, we help each other.”*

“*We are stress-free and it is because of the environment. Here, air is pollution free, food is organic, life is peaceful and we share sorrow of each other, we help in healing any grief and we focus on happiness.”*

“*What is unique and special about Kalasha is that here there is more unity among people and we live by helping each other.”*

“*We all live like a family, there is no hatred between us, no religious conflicts with neighboring communities and we believe in sharing.”*

“*It's (Kalasha) a very peaceful religion, we all live with love here and I like it a lot, we have freedom of choice, but we ask from family as well. We believe in sharing and discussing with them but the good thing is that the elders/family do not force us, so yes this is the reason that we are strong against psychological problems.”*

“*There is a sense of sharing and emotional sharing which is like you are not left alone to suffer something so in this sharing culture the chances of developing something psychologically are limited.”*

The young Kalashas are certain about their community's well-being as they considered their community generally and Kalasha youth specifically, having less chances of developing mental health problems and they attributed their mental well-being to their culture of sharing and connecting with each other. This shows that young Kalasha tend to have high cohesiveness and congenial interpersonal relationships. This theme of intra-communal bonding and sharing reflects the microsystemic approach, if we look at the participants' quotes in the context of Bronfenbrenner's systems. Also, the above quotes refer to the mesosystemic interactions between Kalasha youth and the neighboring communities and we can ascertain the intimate kinship patterns, yet autonomous practices of Kalasha family.

#### Kalasha festivals and traditions

The Kalasha celebrate three major and a few small festivals in a year (41). Every member of the community takes part in these festivals regardless of gender or age. These festivals are mostly considered a way of paying tribute to the nature and gods. They are meant for the well-being of the community and also hold very significant value in Kalasha culture and tradition. The significance of Kalasha festivals are depicted through the following verbatim quotes of the participants:

“*There are 3 major festivals in Kalasha, one is celebrated in December during winters, another is celebrated in Spring in May, that is called ‘Chillamjosh’, and the third one is celebrated in October, mostly in Autumn. There are many other small festivals, but mainly these three are the major celebrations. So in festivals we do get together and dance together and pay tribute to nature, celebrations makes us happy and resilient.”*

“*Traditional celebrations are in order to remember who we are as a people, like the gatherings show a bonding. So the ‘festivals’ that they call it in the print media, are actually not some kind of a drinking parties, or dancing shows. It's not for entertainment. It's a part of a tradition for ancestral culture, this falls into the category where there is a winter, spring and autumn.”*

“*We do preparations from a couple of months for festivals and it's a matter of happiness, we enjoy a lot. Especially the May festival ‘Chilamjosh.’*

“*When festival starts on 3rd May, people with new born babies get together at a place called Jazda Khana, then all mothers place flowers there and an ancient shawl is also tied there, and they also carrie baskets full of walnuts and mulberry. Then they move upwards from Jazda Khana, and from there one man pours milk on new born babies, people have different concepts about this ritual, some says that it's for prosperity and some says that after this babies do not weep much. After that fruits (walnuts, mulberries) are distributed among all and then people go to a dancing place and dance there in groups.”*

These festivals are part of the tradition; have something to do with Kalasha's ancestral and traditional beliefs and customs; and are not some random parties or timeout from the routine. Rather these festivals are well planned and organized events with an objective and are a celebration of new seasons.

#### Purity concept

The ónǰeṣṭa and prágaṭa are a symbolic system of the Kalasha. In past literature, these terms are used in place of “pure” and “impure.” This polarity has semantic meanings which were illuminated by the participants in the given below key quotes:

“*The division of pure and impure instils hope for us to go about ónǰeṣṭa (pure) more, to be connected with our ancestors and with our inner self which helps us become stronger.”*

“*Bashali is a place separated from our houses, located at the down side, for women to stay during menstruation and child birth period. During these periods we are considered as impure. Every month.”*

“*We stay there for a few days and as there is no domestic work we have to do, it's a kind of rest period for us, and even the food is delivered there from our houses. I think it's a good break time from house chores and to contemplate and serves as an opportunity to deal and overcome any stressors.”*

Here our interest in ónǰeṣṭa is to understand how this concept of purity acts as a buffer against psychological distress and contributes to well-being. The research participants used this concept to explain how women during their menstruation shift to Bashali and the women considered and accepted this practice as something that gives them an opportunity to rest and have time for contemplation. The participants also believed that the division of ónǰeṣṭa and prágaṭa gave them strength as it provides them opportunities to connect with one's inner self and with their ancestor's spirits. This reflects their philosophical inclinations toward existential domain that they believe in a spiritual world and have an understanding of connecting with inner self.

#### Behavioral practices of happiness and cognitive patterns

From the social constructivism perspective, every individual constructs a unique reality depending on his/her phenomenology and lived experiences. Here, in illuminating on the concept of happiness, Kalasha youngsters, as a whole group seem to hold similar beliefs about the shared value of happiness. The young Kalasha's cognitive schemas tend to be based upon cultural upbringing and predisposition to the concept of celebrations and happiness. The schematic practice of happiness is manifested through behavioral components as well. This can be comprehended by the explanation of the mentioned quote, where the participant explained how the Kalasha youth are persistent in practices of happiness during times of grief.

The figure-ground relationship of happiness can be best explained through the example of their death ritual, where the concept of happiness *per se* may become ground and the sorrow is the focus or figure, however; in all other social interactions and actions, the concept of happiness is a central element. Savoring refers to attending to and enhancing positive experiences and is the use of thoughts and actions to increase the intensity and duration of positive experiences (42). The young Kalasha's formulation of happiness seems well versed with savoring as their positive focus on happiness, through the cognitive element and physical actions of participation in festivities and celebrations. The savoring of Kalasha youth through behavioral and cognitive patterns again reflects the Bronfenbrenner's macrosystem as this disposition of happiness is inherited and enrooted in their tradition and culture, attitudes and belief system. The young Kalasha give utmost importance to their macrosystem as well as microsystems. Some examples of these behavioral practices in participants' words are given as follows:

“*Our festivals and preparations for festivals make us happy, we do dance, chant and sing with instruments i.e., drum play. It's all happiness and it is the practice of happiness.”*

“*You can say that we are happy because we share our grief and here people support each other and believe in living happily.”*

“*Let's take an example of the death ritual here. It is indeed an expression of sorrow, as the family of that departed person is obviously in grief. However, it appears as an expression of happiness as we dance and sing for the death ritual. Some people might have thought that it's wrong to dance on death….but…in us we think that it's our ritual and done by our ancestors …so we have to follow and by doing this apparent practice of happiness we promote wellbeing and happiness in long term.”*

“*Kalasha are considered as a happy community and the reason is that they are involved in happiness as well as sorrows of each other.”*

“*Even in death we try to find happiness, as we celebrate that sorrow (of death), we do chanting on his (the one who died) name and feel satisfied Actually, our goal of life is to be happy and to be happy upon God's will. This practice of happiness ritual in the times of sorrow reminds us that we've to stay happy in any situation.”*

An embodied practice of happiness emplacing cultural horizons of understanding reflected through “festivals and preparations for festivals make us happy.” Here participants' quotes are well related with savoring—a concept of positive psychology.

### Perceived causes of mental health issues

#### Biological and psychosocial causes

The Kalasha youngsters seem to have an adequate understanding of the etiological factors of stress as well as the genetic vulnerability for psychological problems. The stress vulnerability model (43) seems to be applicable here which identifies stress and biological vulnerability as two important factors contributing to mental health problems and the Kalasha youth also reflected upon these two. Furthermore, one participant shared the psychosocial element of mental health problems with an example of a girl who converted to Islam. The argument that the participant wanted to build reflects the socio-political pressure from the neighboring communities.

“*Yes I know the major cause of mental health issues is ‘stress’ let me share with you an example of a young Kalasha girl who converted to Islam last year. She was not mature and was under the age of 18, so she went to live with a Muslim family. Her parents did many efforts to bring her back home but failed. Soon after that, her cousin went to meet her and found her in stress, and she shared with her cousin that she unintentionally changed her religion. Therefore, her cousin brought her back home, but after that, an angry mob of Muslim people came and forced her to go with them. Her family members spoke to that group and tried convincing them not to force her to go with them when she doesn't want to go with them, and to resolve the matter through dialogue. Unfortunately, they did not listen and attacked her house with stones, until police dispersed the mob by using teargas. Resultantly not only that girl but her cousin who brought her back was also traumatized and now have panic attacks very often.”*

Where we discussed earlier about the importance and congenial microsystem organizations of Kalasha, this theme illuminate another side. This shows that Kalasha youth have to face challenging scenarios in their mesosystem, especially when their microsystems of Kalasha family and neighboring communities interact.

#### Environmental

If we see participants' quotes (as given below) with respect to the environmental causes of mental health problems and also relate these quotes with what the participants discussed earlier i.e., stress and biological vulnerability, it will be just to conclude that the Kalasha youth hold a belief similar to the diathesis–stress model.

“*But now genetically modified crops are coming in, destroying the local breeds of seeds that have been there for thousands of years, fertilizers have been introduced. Even, I have seen the cases where people using this pest thing, and houses being sprayed, which is causing diseases, also mental diseases.”*

“*Mental health as well as physical health both issues are increasing day by day, have you seen? Because the farmers have started using sprays to kill the insects, these sprays contains medicines and contaminating natural things like vegetables, fruits etc. The plants absorbs that medicine and then we eat those plants, we feel like we are eating organic food but in reality, that food is a product of medicines and different sprays. As a result our body organs became weak and it may also effect psychological health.”*

In case of the Kalasha youth, they look upon the clean environment of Kalasha valleys as an important factor for wellbeing, as participants described the conducive and healthy environment of Kalasha, a significant factor acting as a buffer and enhancing resilience against psychological problems.

#### Shamanic treatment

The Kalasha youngsters have a strong belief in their traditional spiritual practices for curing health related problems. We termed this theme as shamanic treatment, although the past literature recommends that it is more appropriate to say that shamanic elements are present in Kalasha culture rather than calling it a full-fledged shamanic institution (5). The practices of treatment are similar to shamanic treatment where the Kalasha de'har/shaman goes into a trance and focuses his/her gaze on the ónǰeṣṭa sphere and provides the remedy after assessment. The “istongus”, as reported by the participants is sacrificing a goat and distributing its meat after sprinkling the goat blood on the patient's forehead. The concept here is the charity/sacrifice which is somewhat similar to the traditions found in other religions, especially Abrahamic traditions. The participants described about this indigenous treatment method in the following quotes:

“*The treatment is called ‘Istongus’ in our language, in which we sacrifice a goat and then blood of that goat is sprinkled on the forehead of the patient. This is done by a specialist, for example, there is a woman who is an expert in this. Actually that blood is a sacrifice for God in order to get cured. It can be effective for any issues….like severe illness, it can also be done for persisting fever, mental issues like among my friends if someone is in stress which is beyond their control they may opt for this method, as it is a successful way to deal with such issues. She will touch the patient and will automatically get to know about their ailment. As for further accurate assessment, she will burn some ring type bangle on the fire and get to know the exact cause/reason. Afterwards she would chant for their relief and also suggest to do charity for cure and it is quite effective.”*

“*There is certain element of strong belief in fairies, so if somebody behaves in a certain way or they see that there is some kind of mental issues, then they try to seek Shamans and they try to seek people who make Ta'awiz. So, Kalasha who is suddenly sick or something they will seek every way possible like they will go to the doctor if that is possible. It all depend on their resources, if someone can afford they may take patient to Islamabad, if they couldn't, they would then seek the Shaman treatment or the Molvi or the people who deal in making ta'awiz.”*

The unique quality of Kalasha youth and their spiritual tradition is the flexibility and openness to all the available treatment options. The very next theme (i.e., ta'awiz or amulets) reflects the young Kalasha's openness and acceptance of other traditions. It is not the case that they are not aware of medical options, especially the group under study constitutes the youth of Kalasha, who are increasingly aware of the evidence based practices of treatment. The spiritual options are chosen considering their easy access, respecting the beliefs and, following the advice of their elders and ancestors. The shamanic treatment reflects the Bronfenbrenner's microsystem, if we consider shamanic experts as a microsystem organization.

#### Ta'awiz (amulets)

The Kalasha have been living in a close proximity with the Muslim community. In the recent decades, the Muslim population has settled closely to the main entrance of the Kalash valleys. The Muslim preachers come all the way from Punjab and Khyber Pakhtunkhwa provinces for preaching purposes and try to convert Kalasha. The conversion of Kalasha is also an issue reported; whereas some participants believe it is a forced conversion, others think that they convert with their free will and without any pressure. Here, the focus is not to explore their conversion process but understanding their perspective in terms of consulting Muslim clerics (Molvis) for seeking cure of physical or mental health problems. The Kalasha participants shared their views in the following statements:

“*People believe in amulets for cure of mental health, but our generation does not believe in them. For example, I know someone who has epilepsy and is seeking treatment with amulets in order to treat epilepsy! They do not consult the doctor. If anyone faces any mental health issue, they use amulets only and get those amulets from Muslims. Kalasha believe that amulets will cure them….A girl in my community had some mental health problems, her family consulted Molvis for amulets and Qazis for her spiritual treatment, they also went to Peshawar and Islamabad but that problem is not cured yet.”*

“*If one takes tension, the solution according to me is using amulets, with amulets a person can get health. The Kalasha community believes in amulets, because are helpful to alleviate tension. We believe that God gives health through amulets, and obviously access to a hospital is not easy as it is far away, whereas amulets are available here, so we use them mostly. We take patients to Molvis for amulets, we are not aware of the verses he writes in that amulet as we are not supposed to open that. People prefer to go to Molvis and take amulets rather than going to doctors and hospitals. Sometimes when we take a patient to a Molvi, he may refer or recommend taking the patient to the medical doctor, when he believes that the case is not of a spiritual nature.”*

The practice of making ta'awiz (amulets) is common in some sects of Islam and among the Sufi orders. The Kalasha youth seem to have been influenced by the ritual of getting amulets from Muslim Molvies. This phenomenon is an emerging trend in Kalasha, which was not present in their traditional practices. This phenomenon emerged with the conversions as the converted Kalashas have faith in the healing powers of Quranic verses and they share with other non-converted Kalasha. This has been evident largely in more remote valleys of Barir and Rambur as compared to Bamburat valley. The valleys of Barir and Rambur are in a constant process of acculturation, whereas, Bamburat valley's Kalasha people seem to be resistant to the process of this change and want to conserve their cultural heritage.

#### Communal sharing and problem solving

The homogenous structure of the Kalasha community portrays Kalasha as one community. It is a small community spread over three rural valleys (i.e., Barir, Rambur, and Bamburat). Each valley is located at a distance and approach to these valleys is not very easy as no proper roads are constructed and one has to put the vehicles on the mountain tracks in order to reach every valley. The distance between Bamburat valley and the Rambur valley is 1,640 m. There is a pass connecting the Birir and Bamburat valleys at around 3,000 m. The Kalash towns in each of the three valleys are situated at a height of around 1,900–2,200 m (44, 45).

Besides the difficulty, all the three valleys of Kalasha are connected to one another during times of happiness and sorrow. They have a culture of sharing and caring. This empathetic culture was fostered through their tradition and their ancestor's emphasis on sharing and mutual respect. It wouldn't be wrong to say that each young Kalasha has inherited the positive traits of mutual respect, sharing and generosity. The example of the death ritual in the following quote reflects how the young Kalasha contribute financially and emotionally in order to retain their traditions and rituals.

“*In order to understand the culture of sharing, let's take an example of the ritual of death, which is very costly here, because when someone dies, people from all the Kalasha valleys come here. The mourning family has to slaughter 30 to 50 goats as a death ritual and to feed all the community gathered for funeral ceremony. The goats are very costly and also they have to arrange a large quantity of cheese in addition to goats, it is again challenging. So if that family doesn't have goats or money to purchase goats, people actually help that family to manage the expenses in this difficult time, every member of Kalasha offers help; they give and do a lot of charity and it is on a volunteer basis with no obligation, it's totally up to the person helping to contribute whatever he/she can. When someone dies, all people sit together in a community meeting and decide what to contribute. This culture of sharing is not limited to death rituals only, but we practice the same for any other problem.”*

Once again, we see the relevance of the Bronfenbrenner's macrosystem as a belief system holding the values of sharing and caring reflects the macrosystem.

#### Medical treatment

The Kalasha youngsters have explained about the medical treatment options in the following quotes:

“*Kalasha goes to hospitals for the treatment of mental health issues, mostly they go to Chitral. A girl had some mental health problems, she was taken to the hospital; there she stayed for two months but she is still in the same condition. Sometimes she has panic attacks. Her medical treatment is continued. Most of the times, we go to doctors but if remain uncured, then we go for spiritual treatment. If someone is suffering from mental health issue, we do consult doctors to know about diagnosis. As far as I know, mental health issues are not common here, people are mentally healthy here. Only in extreme cases, people consult doctors and go to the major city for treatment. But for minor issues like anger, temper tantrums, anxiety and stress people seek communal sharing and problem solving and use local practices.”*

“*Some people (Kalasha) go to doctors. In my opinion, doctors treat psychological issues better as they are specialized in the field so they do understand the nature or complexity of the problem, whereas, do not work. But sometimes people do not get cure from doctors even. I request for the government to appoint good doctors and better facilities in our hospital like MRI, ECT and other medical treatments etc. to stop this culture of amulets.”*

Kalasha youth have an understanding of the medical causes of mental health problems and they realize that the medical treatments should be considered but they are left with other treatment options due to various limitations and barriers in accessing the medical facilities especially the psychiatric facilities which are available only in main cities. Also, there is awareness that consulting faith healers and using amulets are not very effective options as one participant appealed and highlighted the need to appoint specialized doctors and equipment in their local hospital in order to stop the trend of consulting faith healers. The medical option, in some cases, is the last remedy when the condition of the patient doesn't improve from other options. This is because the medical option is a bit difficult to approach considering the peculiar geographical location of the Kalasha valleys.

#### Herbal methods

The Kalasha youngsters tend to believe that organic food can be a good protective factor against physical and mental health problems. The Kalasha youth also illuminated about using herbal methods as shown in the following excerpt:

“*There are medicinal plants in Kalasha valleys, which can cure problems related to physical and mental health. These plants have curative properties for both humans and livestock. We have a specific plan, which is used for the treatment of fertility issues and for ease in birth.”*

They have strong belief in the effectiveness of medicinal plants and traditional practice of using herbs.

## Discussion

The aim of the current study was to understand young Kalasha's lived experience and sense making of mental health and psychological resilience. The aim was also to explore young Kalasha's cultural protective factors against mental health issues and to make sense of Kalasha's world and their perceived causes of mental health problems and their help seeking behavior/ preferred treatment options. The themes discussed in the results section shed light on Kalasha's perspectives.

The relationships within any society or group of people are defined by social capital. The most widely used definition of social capital in health sciences is given by Putnam (30). According to Putnam, there are five main aspects of the social capital: (1) community networks, voluntary, state, personal networks, and density; (2) civic engagement, participation, and use of civic networks; (3) local civic identity—sense of belonging, solidarity, and equality with other members; (4) reciprocity and norms of cooperation, a sense of obligation to help others, and confidence in return of assistance; (5) trust in the community (30). According to the theory of social capital, the many components in this concept are a behavioral/activity component (also called structural social capital, e.g., participation) and a cognitive/perceptual component (also called cognitive social capital, e.g., trust) (46).

Kalasha's structural and cognitive social capital are reflected from the themes of intra-communal bonding and correspond to connections among individuals who resemble each other such as individuals in Kalasha community or individuals of the same socioeconomic status (also known as bonding social capital). The evidence of a relationship between social capital and mental health was shown in a systematic review. This reveals the presence of a negative relationship between the individual level cognitive social capital and common mental disorders (47). Furthermore, the past research (48) has shown better health outcomes in the communities where there is higher involvement of people in community activities as compared to those communities where there is lower civic engagement. Kalasha surely seem to have high involvement in community activities as also evident from the themes of Intra-communal bonding and sharing, and Kalasha festivals and traditions.

The research also showed a positive correlation between social participation (such as cultural festivals) and wellbeing. Past empirical evidence suggests: community festivals are associated with opportunities for community cultural development (49); festivals are building blocks for communities and encourage ethnic understanding within society (49–51), festivals safeguard and celebrate local traditions, history, and culture, or can be used as an approach to spread a destination's lifecycle (52). Engagement, a measure of a person's purpose in life, is a chief element in positive psychology. Research has linked engagement with various health outcomes, both physical and psychological (53). Seligman emphasized the significance of well-being, and gave forth five quantifiable measures to reflect this construct; referred by the acronym PERMA: Positive Emotion, Engagement, Positive Relationships, Meaning, and Accomplishment (53). According to Seligman, higher engagement is associated with improved well-being and a meaningful life (53).

There is a role of community-based festivals in improving mental health and wellbeing at the individual, organizational, and community level, however, there is limited research investigating the link between community festivals and wellbeing. The festivals are shown to bring about meaningful and fulfilling social interactions beneficial for health and wellbeing at the individual as well as community level (48, 54). This is well related with the theme of Kalasha festivals and traditions and Behavioral practices of happiness and cognitive patterns, where they discussed in detail how good they feel planning and celebrating these festivals and how it becomes a source of happiness for them and serves as a protective factor against psychological problems.

Also, referring to Kalasha's concept of purity, the positive meaning of the term ónǰeṣṭa doesn't mean that prágaṭa is associated with sin or the devil (5, 55). Also, like our understanding of the “pure/impure”, these two poles of ónǰeṣṭa and prágaṭa are not related to sexual morals, cleanliness or devotion (5, 18). Nowadays, as a result of living with Muslims and influence of Islam, Kalasha people talk about paradise and hell and occasionally relate the myth of Adam and Eve. However, the true Kalasha tradition has no concept of after life, idea of a prize or a punishment in the hereafter—a view found also in the Veda (5, 56) and in early Rome (5, 56).

In case of violating the rituals, apology is sought ritually and reward for praiseworthy acts is granted here on Earth, with the respect they give and the endless memory posterity will keep of the person who performed them. The idea of immortality does not seem to be linked to something that survives death, like the Christian/Islamic soul, and it is not related to another world. However, it has to do with being remembered in this world. According to the Kalasha, remembrance is kept alive through the songs and panegyrics rejoicing the feats of the ancestors, continually carried out at every festival, and through the wooden statues (effigies) engraved for those who in their lives have achieved commendable deeds (5). In discussing the Kalasha's concept of purity in light of literature, we cannot move beyond the ground breaking research of Kohlberg, where morality was conceptualized in terms of harm and justice, which included values relating to individual rights, fairness, and personal autonomy (57). Nevertheless, moral judgments can encompass other domains besides harm and justice such as loyalty to group, respecting authority, and, conserving purity and sacredness. Kalasha forms the best example of conformity, respecting authority, loyalty to their ethnic group and Kalasha's efforts for conserving purity and sacredness are evident from their concept of ónǰeṣṭa and prágaṭa.

Lastly, the domain of purity comprises values and principles oriented at safeguarding the sacredness of the body and soul. It is the belief in the purity domain that people ought to be, in their bodies and minds, clean, chaste, self-controlled, and spiritually pure and should endeavor to lead life in a sacred, divine way (the belief in deity is not a necessary requirement). From the stance of purity, to reject polluting forces or hedonistic pleasure, to purify the soul, and to behave according to the “natural order” is righteous. It is immoral to act in a way that is “self-polluting, filthy, profane, carnal, hedonistic, unnatural, animal-like, or ungodly” (58–60).

We can understand the theme of Kalasha's behavioral practices of happiness and cognitive patterns by relating it with the “savoring” concept of positive psychology. It is believed that savoring, as the set of cognitive or behavioral strategies, is a monitoring process impacting the association between positive events and a person's positive affective responses to these incidents (61, 62). Savoring is defined as a mechanism whereby individuals engage “to attend to, appreciate, and enhance the positive experiences in their lives” (42).

According to Bryant and Veroff, there are many cognitive and behavioral strategies of savoring that are involved in enhancing and extending positive experiences, including sharing the event with others, behavioral expression, counting blessings, self-congratulation, memory building, and sensory-perceptual sharpening (42). Another concept resembling savoring is view of “capitalizing” given by Langston (63). Capitalizing is defined as constructively understanding positive incidents (63). The past empirical evidence has shown the effectiveness of savoring as a mechanism of positive affective regulation that maintains and deepens positive emotions (42, 64, 65). There is a mediating and/or moderating role of savoring in the relationship between positive events and happiness (42). Now as we have explained and related the findings of psychological resilience of the current study with the past literature, in next section we will discuss the second major component of the current study i.e., mental health conceptualization of Kalasha.

The first theme in the perceived mental health causes of Kalasha was *Biological and psychosocial causes*. The past empirical studies have suggested three models regarding the causes and treatment of mental health; the supernatural, biological and psychosocial models. The people in developed countries have more inclination toward and accept biological and genetic aetiologies of mental health issues (66–68). But surprisingly the minority community of Kalasha from a developing country seems to have an adequate understanding of biological and psychosocial causes of illness. The biological model suggesting chemical imbalance, hereditary and genetic causes of illness as well as the psychosocial model linking mental health problems with issues such as stress, trauma, abuse, and family and relational conflicts have received considerable empirical support (69–74).

The literature has highlighted that the people in developing countries have more inclination toward and accept supernatural forces, evil spirits and spiritual aetiologies of mental health issues and choose indigenous treatment modes (75–79). Despite the fact that Kalasha do have an understanding of biological and psychosocial causes but they also seems to believe in the supernatural causes of mental health problems. A meta-synthesis study revealed the perception of people assigning mental health issues to blessings and spiritual connection with God and getting special attention from nature (80, 81). Moreover, the belief on spiritual treatments as the preferred mode of treatment was present in the past studies (80–83).

The supernatural model of mental health issues has received support from a few studies (82, 83). However, according to a few research studies, traditional healers are shown as providers of effective psychosocial intervention; relieving distress and improving mild symptoms in common mental issues such as depression and anxiety (28, 75, 80). Nevertheless, there is limited evidence supporting the effectiveness of traditional healers in managing severe mental health problems such as bipolar, psychotic disorders, or obsessive-compulsive disorder (28, 75).

There is a possibility that the traditional healing could bring more harms than good. It is likely that individuals with mild issues and positive expectations seek subjective benefit from joining their chosen traditional or spiritual healers. There is existence of value in those individuals seeking and finding meaning in attending the traditional healers. The individuals find the traditional healers beneficial despite absence of any betterment in symptoms. There is ambiguity about how these traditional healers work, though exhaustive regular social interventions usually attain better results as compared to short-termed single interventions. The same concern is also raised by few of our study participants who seem skeptical of the traditional and supernatural methods.

There is a call for a more holistic care and potential synergies for mental health, if an association between healing systems can be enabled for the individuals having cultural and spiritual beliefs contrary to conventional psychiatry (84). Our participant discussed about herbal methods as treatment of health related problems. The herbal medicine—a famous complementary and alternative medicine (CAM) is used worldwide (85) in treatments of a range of health issues which may include common mental health problems (86). Lastly, we will discuss how our chosen theoretical framework of Bronfenbrenner's ecosystems can be applicable in the context of this study for Kalasha. The same has been discussed in the result section with the themes where Bronfenbrenner's system seems applicable.

Interpersonal relationships comprise the microsystem and mesosystem. When Kalasha discussed about strong interpersonal relationship, communal bonding and sharing and celebrating their festival, this actually refers to their strong microsystem and macrosystem. The microsystem recognizes a person's face-to-face interaction with others such as his/her family, school, and peers (2, 86, 87). However, the mesosystem deals with multiple microsystems that operate side by side (1); for instance, a Kalasha celebrating their traditional festival after consulting with their traditional/religious elders who decides the date of the festival.

The macrosystem—comprising the overarching worldview of a community and culture—investigated the uniformities within the subculture, existing belief system in the culture, or ideology that had been formulated from the lower order systems (87). The macrosystem is the most relevant system in the case of Kalasha as their lives are guided by their traditional beliefs and they valued a lot to their ancestral traditions and promoting their communal bonding. This model enlightened the researcher that a person's ecological mechanisms occur in a dynamic manner with interplay of various factors (27, 28).

### Strengths, limitations, and future directions

This study established the usefulness of the Bronfenbrenner's bioecological model in recognizing various resilience factors in a developing individual's environmental context, but the focus on the exosystem and chronosystem was less in Kalasha's context of this study. Only one participant's example illustrated in the theme of psychosocial causal factors reflects the relevance of chronosystem where she commented about the societal and geopolitical pressure and environment surrounded by the Kalasha. Additionally, this model offers a baseline of characteristics to acknowledge the developing individual's protective factors. This study explores the intricacies and complexities regarding mental health existing in the environment of the Kalasha youth.

The limitation of the study includes the broader scope of the study as this study is one of the first initiatives to conduct empirical study on Kalasha from the psychological/mental health angle, therefore there was no past psychological literature available on Kalasha. Hence, we decided to frame this study with the broader scope of mental health rather than operationalizing the specific variables representing mental health. Secondly, despite the Bronfenbrenner bioecological model's identification of protective factors, there is a possibility that any other possible resilience factor may have overlooked, which we (researcher and participants) couldn't bring in during the interview.

Future studies can be planned on Kalasha to explore the relationship between their savoring practices and psychological wellbeing and happiness as well as on their acculturation process. In any future study, it is recommended to include local collaborators who belong to the Kalasha community in order to promote decolonizing research.

## Conclusion

The present study provides the contribution to the field of psychology by identifying cultural protective factors and mental health perceived causes and treatment options used by a community which is struggling for its survival and for conservation of its cultural heritage. The resilience factors identified through this study can be used in clinical settings for devising management plan for fostering resilience and to deal with psychological distress faced by similar indigenous communities in South Asia. The findings of this study highlight Kalasha's lack of access to quality higher level mental health care for severe cases of mental health problems, and the disassociation between medical and indigenous constructs which jeopardizes effective care.

## Datasets are available on request

The raw data supporting the conclusions of this manuscript will be made available by the authors, without undue reservation, to any qualified researcher.

## Author contributions

All authors listed have made a substantial, direct and intellectual contribution to the work, and approved it for publication.

### Conflict of interest statement

The authors declare that the research was conducted in the absence of any commercial or financial relationships that could be construed as a potential conflict of interest.
